# Drivers of diversity within and between microbial communities during stochastic assembly

**DOI:** 10.1098/rsif.2025.0329

**Published:** 2025-11-05

**Authors:** Loïc Marrec, Claudia Bank

**Affiliations:** ^1^ Institut für Ökologie und Evolution, Universität Bern, Bern, Switzerland; ^2^ Swiss Institute of Bioinformatics, Lausanne, Vaud, Switzerland

**Keywords:** microbial ecology, community assembly, dispersal, richness, dissimilarity, diversity, microbial communities

## Abstract

No two microbial communities share the same species richness and abundance profiles. Experiments have shown that the assembly of new microbial communities from the same environmental pool is sufficient to generate diversity within and between communities: when microbial dispersal is slower than division, communities exhibit low richness but high between-community dissimilarity; when dispersal is faster, richness increases while dissimilarity decreases. Here, we study a minimal stochastic model that recovers these empirically observed assembly regimes. Our mathematical framework yields explicit expressions for the abundance fluctuation distributions across low-, intermediate- and high-dispersal regimes, providing a quantitative lens on microbiome assembly. We derive analytical predictions for the bimodality coefficient that quantifies the transition between assembly regimes, which appears as a robust metric to predict community richness and dissimilarity. Additionally, we highlight the mean relative abundance as a complementary metric sensitive to differences in microbial traits (e.g. dispersal or division rates). Applying these metrics to experimental data indicates their practical value for the rapid identification of assembly regimes and trait asymmetries. Overall, our study provides general predictions about how stochasticity, timescales and microbial traits influence both within-community diversity (richness) and between-community diversity (dissimilarity) during the assembly of new microbial communities. Our work thus contributes to a better understanding of the factors driving variation in microbiome formation.

## 1. Introduction

Microorganisms inhabit marine, terrestrial and host-associated ecosystems. In addition to being everywhere, they are present in large numbers. There are $10^{29}$ bacteria in the oceans, $10^{9}$ in a teaspoon of soil and $10^{11}$ in a gram of dental plaque [1]. Moreover, microbial communities play a critical role in numerous biological processes. This sparks great interest from basic and applied science in understanding their structure and function. Recent advances in genomic and metagenomic sequencing have shed light on the co-occurrence of several microbial species within individual communities, which often build complex multi-species ecosystems. It is increasingly evident that even microbial communities found in seemingly similar environments often differ in species richness and abundance. The origins and drivers of this vast diversity within and between microbial communities (i.e. richness and dissimilarity) remain to be identified. Comprehensive theoretical models are needed to quantify the processes driving these diversity patterns.

The growing interest in deciphering the mechanisms underlying such diversity has led to numerous bottom-up experimental studies. Some of them suggest that diversity emerges in the early stages of microbial community assembly [2–4]. Indeed, many large microbial communities assemble from scratch when a new micro-environment is formed. For example, volcanic eruptions create sterile environments ripe for new microbial assembly, such as hot springs, fumaroles and lava tubes [5]. Moreover, many organisms do not inherit their parents’ gut microbiota and, thus, are born with germ-free guts that are populated by microbes only after birth (e.g. *Caenorhabditis elegans* [6], *Drosophila melanogaster* [7]), making community assembly a potentially important stage in the emergence of diversity. Other organisms, like humans, inherit a small fraction of their parents’ gut microbiota [8]. In addition to diversity within individual communities, gut microbial communities also exhibit high diversity between communities. For example, substantial variation in microbial community structure (i.e. composition and abundances) is observed among hosts of the same species [9–11]. Even monozygotic twins were shown to have high within-twin-pair microbiome diversity [12,13].

To identify the key drivers of diversity within and between microbial communities, experimental studies increasingly use a *bottom-up* approach to assemble new communities. Assembling microbial communities under controlled conditions, such that experimentalists can systematically manipulate factors such as the species composition of the microbial pool, helps quantify how these factors influence the emergence and maintenance of diversity within individual communities. Additionally, when scaled across multiple microbial communities, these experiments provide insights into how variation in microbial community structure arises. Such bottom-up experiments are essential to isolating the mechanisms that govern diversity patterns, advancing our understanding of microbial ecology. For example, Vega & Gore [2] carried out an experiment involving initially germ-free *C. elegans* worms fed on *Escherichia coli* bacteria. They showed that community assembly in the intestines of hosts that are isogenic, isolated and fed on the same bacterial pool produces substantial inter-host heterogeneity [2]. Their results highlighted that the early stages of microbial communities are not only driven by deterministic mechanisms, such as selection, but also by stochasticity. However, the importance of random events in shaping microbial communities relative to deterministic mechanisms is contentious [14]. There is therefore a strong need for mathematical models accounting for stochasticity to better understand diversity patterns.

Here, we build a stochastic model inspired by previous work from Houchmandzadeh [15] and Vega & Gore [2], which accounts for two microbial features/traits: the rate of microbial dispersal from a pool to communities and the division rate. By analysing timescales of dispersal and division, we recover distinct assembly regimes that give rise to different diversity patterns, consistent with the experimental and simulation study of Vega & Gore [2]. Importantly, we derive analytical predictions for species richness and abundance fluctuation distributions, validated by numerical simulations. In addition, we investigate how trait differences shape within- and between-community diversity across assembly regimes. Our quantitative analysis identifies two key metrics, the bimodality coefficient and the mean relative abundance, which determine the assembly regime and quantify trait differences. To demonstrate their practical application, we apply these metrics to experimental data from Vega & Gore [2]. Overall, our study quantifies how stochasticity, timescales and traits impact diversity within and between communities during the assembly of new microbial communities, allowing for a better understanding of microbiome formation.

## 2. Model and methods

### 2.1. Community assembly model

In this work, we investigate the assembly of microbial communities. These communities are initially germ-free, and we assume that microbial traits are identical across communities. This set-up reflects certain experimental designs, particularly those involving clonal hosts such as worms or flies, where each host presents the same ecological environment to microbes.

**Figure 1.**
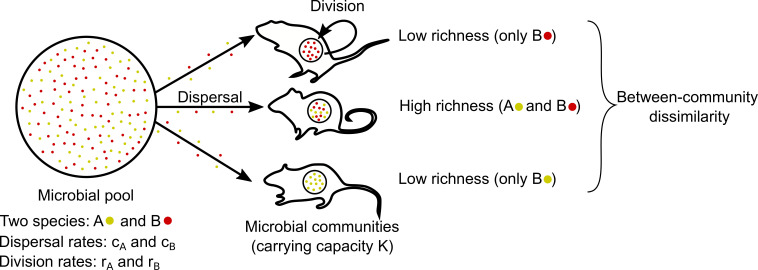
Illustration of the model—microbial community assembly. The environmental microbial pool contains two species, A (yellow) and B (red). These microbes disperse from the pool into local microbial communities at rates $c_{A}$ and $c_{B}$ , and divide within these communities at rates $r_{A}$ and $r_{B}$ . Once the carrying capacity K of the microbial community is reached, we determine whether the two species co-occur (high richness) or do not co-occur (low richness). We compare the composition of several communities to quantify between-community dissimilarity.

We focus on an assembly process driven by two events known to influence microbial community dynamics: dispersal and division [16,17]. As illustrated in figure 1, microbes disperse from a fixed microbial pool into communities and subsequently divide within them.

The microbial pool is fixed and consists of two species, A and B, each defined by two key traits. We consider more than two species in the electronic supplementary material, section S5. The division rates of the two species are denoted by $r_{A}$ and $r_{B}$ . The difference between these rates determines the selection coefficient $s=r_{A}-r_{B}$ , which indicates whether species A is favoured ( s>0 ) or disfavoured ( s<0 ) by natural selection within a community. Their dispersal rates, $c_{A}$ and $c_{B}$ , may differ due to unequal abundances in the microbial pool or differences in their ability to populate a community.

Community size, denoted by $N=N_{A}+N_{B}$ , can grow over time but is bounded above by a fixed carrying capacity K , which could reflect, for example, physical space limitations. Microbial growth follows logistic dynamics, meaning the *per capita* division rate of species A is $r_{A}(1-N/K)$ , and similarly for species B with $r_{B}(1-N/K)$ . This formulation assumes there are no inter-specific interactions beyond neutral competition for space, as both species are constrained only by the total community size relative to the carrying capacity. The model is also adaptable to other forms of density dependence, such as Gompertz or Richards growth [18].

We multiply the dispersal rates *c*_A_ and *c*_B_ by the term (1−N/K) . This formulation reflects the reduced probability of successful establishment of incoming microbes when the community is near saturation. As N approaches K , available space or resources diminish, making it harder for new dispersers to establish, even if dispersal continues to occur. This approach mirrors the logistic limitation applied to division rates and captures ecological effects such as density-dependent invasion resistance and priority effects. We verify in the electronic supplementary material, section 9, that our main results remain robust when the saturation term is removed from the dispersal rate.

In our core model, we do not include an explicit death rate. Instead, we use logistic constraints on both division and migration through the saturation term (1−N/K) , which accounts for density-dependent limitations as the community approaches its carrying capacity. Importantly, we show in §4.6 and in electronic supplementary material, section S6 that our main conclusions remain robust when explicit death rates are included.

To describe the outcome of community assembly, we focus on the abundance fluctuation distribution, which captures the variability of species abundances across replicate communities subjected to the same underlying processes. This distribution should not be confused with the species abundance distribution, which refers to the distribution of species within a single community. The abundance fluctuation distribution provides a basis for quantifying both richness, an α -diversity metric reflecting within-community diversity and between-community dissimilarity, a β -diversity metric that captures how much communities differ from one another.

### 2.2. Community assembly simulation

We simulate the assembly of a microbial community with a Gillespie algorithm [19,20]. This algorithm generates trajectories of stochastic dynamics whose event rates are known. Our model features four types of events: the divisions of the microbes of species A and B


$$(N_{A},N_{B})\overset{r_{A}\left(1-\frac{N_{A}+N_{B}}{K}\right)N_{A}}{\to }(N_{A}+1,N_{B})\;,$$

and


$$(N_{A},N_{B})\overset{r_{B}\left(1-\frac{N_{A}+N_{B}}{K}\right)N_{B}}{\to }(N_{A},N_{B}+1)\;,$$

and the dispersal of the microbes of species A and B


$$(N_{A},N_{B})\overset{c_{A}\left(1-\frac{N_{A}+N_{B}}{K}\right)}{\to }(N_{A}+1,N_{B})\;,$$

and


$$(N_{A},N_{B})\overset{c_{B}\left(1-\frac{N_{A}+N_{B}}{K}\right)}{\to }(N_{A},N_{B}+1)\;.$$

For each event, the propensity function is indicated above the arrow. The simulation steps are as follows:

(1) *Initialization*. The microbial community starts from $N_{A}=0$ and $N_{B}=0$ .
(2) *Event selection*. The next event to occur is chosen randomly proportionally to its probability. For example, the division of a microbe of species A is chosen with probability $r_{A}N_{A}/[(r_{A}N_{A}+c_{A})+(r_{B}N_{B}+c_{B})]$ .
(3) *Population size update*. The population sizes are updated according to the event selected in Step 3. For example, if the division of a species A microbe is chosen, the population size of species A is updated by $N_{A}\to N_{A}+1$ .
(4) We return to Step 2 until the microbial community is populated up to the carrying capacity (i.e. $N_{A}+N_{B}=K$ ).

In the following, we consider that each stochastic realization of the above algorithm describes the assembly of a single microbial community. Thus, collecting several stochastic realizations is equivalent to simulating the microbial assembly of several communities.

## 3. Formal analysis

### 3.1. Master equation

The assembly of a new microbial community is a stochastic process that involves, in our model, two types of events: dispersal of a microbe from the microbial pool to the community and microbial division. As the microbial community is assumed to be initially microbe-free, every assembly process includes a phase in which the community size is small. During this phase, stochasticity plays a major role, regardless of the carrying capacity. We, therefore, use a microscopic and probabilistic description of the microbial community assembly similar to the model developed by Houchmandzadeh [15], which includes dispersal in addition to division. Our model is similar to that of Vega & Gore [2], neglecting death for mathematical convenience; we relax this assumption in the electronic supplementary material, section S6. Specifically, we analyse a system of equations governing the dynamics of the probability $P(N_{A},N)$ of having $N_{A}$ microbes of species A in a community of size N (and thus $N_{B}=N-N_{A}$ microbes of species B). This system of equations, called the master equation [21,22], satisfies


(3.1) $$P(N_{A},N+1)=\alpha_{N}^{N_{A}-1}P(N_{A}-1,N)+(1-\alpha_{N}^{N_{A}})P(N_{A},N)\;,$$

where $\alpha_{N}^{N_{A}}$ is the probability that the increase in community size by one individual (i.e. N→N+1 ) is due to the dispersal or division of a microbe of species A. This probability is given by


(3.2) $$\begin{array}{lcr} & \alpha_{N}^{N_{A}}=\frac{r_{A}N_{A}+c_{A}}{r_{A}N_{A}+c_{A}+r_{B}(N-N_{A})+c_{B}}\;. & \end{array}$$

As pointed out by Houchmandzadeh [15], this probability does not depend on the type of density dependence. It is thus valid for other growth types (e.g. Gompertz, Richards, etc.; see [18]). Since we assume that the microbial assembly starts from a microbe-free community, equation (3.1) admits as initial condition P(0,0)=1 . Moreover, at the two boundaries of equation (3.1) (i.e. for N=0 and N=K ), it reduces to a one-term recurrence relation. For example, considering the lower boundary N=0 leads to a recurrence relation on the probability P(0,N) of having no microbe of species A in a community of size N , which reads


(3.3) $$\begin{array}{lcr} & P(0,N+1)=(1-\alpha_{N}^{0})P(0,N)\;, & \end{array}$$

the solution of which is


(3.4) $$\begin{array}{lcr} & P(0,N)=\frac{c_{B}}{c_{A}+c_{B}}\frac{{\left(1+\frac{c_{B}}{r_{B}}\right)}_{N-1}}{{\left(1+\frac{c_{A}+c_{B}}{r_{B}}\right)}_{N-1}}\;, & \end{array}$$

where $\left(x{)}_{N}=x(x+1)(x+2)...(x+N-1\right)$ is the Pochhammer symbol. Another way to recover equation (3.4) is to note that $P(0,N)=\prod_{q=0}^{N-1}(1-\alpha_{q}^{0})$ . Similarly, we get an expression for the probability P(N,N) of having only microbes of species A in a community of size N , noting that $P(N,N)=\prod_{q=0}^{N-1}\alpha_{q}^{q}$ *,* which leads to


(3.5) $$\begin{array}{lcr} & P(N,N)=\frac{c_{A}}{c_{A}+c_{B}}\frac{{\left(1+\frac{c_{A}}{r_{A}}\right)}_{N-1}}{{\left(1+\frac{c_{A}+c_{B}}{r_{A}}\right)}_{N-1}}\;. & \end{array}$$

By evaluating equations (3.4) and (3.5) at N=K , i.e. $P_{0}=P(0,K)$ and $P_{K}=P(K,K)$ , respectively, we determine whether both microbial species are present at the end of the assembly process. More specifically, the probability that both microbial species co-occur, denoted by $P_{\mathrm{cooc}}$ , is given by


(3.6) $$\begin{array}{lcr} & P_{\mathrm{cooc}}=1-P_{0}-P_{K}=1-\frac{c_{B}}{c_{A}+c_{B}}\frac{{\left(1+\frac{c_{B}}{r_{B}}\right)}_{K-1}}{{\left(1+\frac{c_{A}+c_{B}}{r_{B}}\right)}_{K-1}}-\frac{c_{A}}{c_{A}+c_{B}}\frac{{\left(1+\frac{c_{A}}{r_{A}}\right)}_{K-1}}{{\left(1+\frac{c_{A}+c_{B}}{r_{A}}\right)}_{K-1}}\;. & \end{array}$$

Note that in our work, co-occurrence refers to both species being present at the end of community assembly, making the probability of co-occurrence a first indicator of diversity.

### 3.2. Very low-dispersal regime

When the dispersal rates are much lower than the division rates (i.e. $c_{A},c_{B}\ll r_{A},r_{B}$ ), the probability $\alpha_{N}^{N_{A}}$ (see equation (3.2)) simplifies to


(3.7) $$\begin{array}{lcr} & \alpha_{N}^{N_{A}}=\frac{r_{A}N_{A}}{r_{A}N_{A}+r_{B}(N-N_{A})}\;. & \end{array}$$

The resulting master equation was extensively investigated by Houchmandzadeh [15] (see equation (3.1)). It cannot be analytically solved if both division rates differ, although some approximations exist (see [15] for more detail). Note, however, that equations (3.4) and (3.5) simplify to


(3.8) $$P(0,N)\underset{c_{A},c_{B}\ll r_{B}}{\approx }\frac{c_{B}}{c_{A}+c_{B}}\;$$

and


(3.9) $$\begin{array}{lcr} & P(N,N)\underset{c_{A},c_{B}\ll r_{A}}{\approx }\frac{c_{A}}{c_{A}+c_{B}}\;, & \end{array}$$

when the dispersal rates are much lower than the division rates. These probabilities indicate that, by the end of community assembly, each community will consist exclusively of either A or B microbes, depending on the species’ dispersal rates. In other words, the very low-dispersal regime leads to a probability of co-occurrence of zero (see equation (3.6)). In addition, it is important to note that community structure in this regime does not depend on division rates and is therefore indifferent to within-community selection.

In this regime, the mean relative abundance of species A, denoted by $\left\langle N_{A}(N)\right\rangle$ , corresponds to the first moment (i.e. the mean) of the abundance fluctuation distribution. It simply satisfies


(3.10) $$\begin{array}{lcr} & \langle N_{A}(N)\rangle =\frac{c_{A}}{c_{A}+c_{B}}\;. & \end{array}$$

### 3.3. Very high-dispersal regime

When the dispersal rates are much larger than the division rates (i.e. $c_{A},c_{B}\gg r_{A},r_{B}$ ), the probability $\alpha_{N}^{N_{A}}$ (see equation (3.2)) simplifies to


(3.11) $$\begin{array}{lcr} & \alpha_{N}^{N_{A}}=\frac{c_{A}}{c_{A}+c_{B}}\;. & \end{array}$$

This simplification allows us to solve the master equation (see equation (3.1)), the solution of which reads


(3.12) P(NA,N)=(NNA)(cAcA+cB)NA(cBcA+cB)N−NA.

Thus, the number of A microbes in a community follows a binomial distribution in the very high-dispersal regime. In this case, the probability of co-occurrence depends on the relative dispersal rates of the two species. Specifically, when the dispersal rates differ substantially, the probability of co-occurrence approaches zero. As in the very low-dispersal regime, community structure here does not depend on division rates and is therefore indifferent to within-community selection. Interestingly, the very high-dispersal regime exhibits the same mean relative abundance of species A as the very low-dispersal regime, i.e.


(3.13) $$\begin{array}{lcr} & \langle N_{A}(N)\rangle =\frac{c_{A}}{c_{A}+c_{B}}\;. & \end{array}$$

### 3.4. Neutral case

Here, we assume that both microbial species, namely A and B, have the same division rate. This neutral case was experimentally investigated by Vega & Gore [2] using two bacterial strains that only differed in their fluorescent label (YFP and dsRed). Such neutrally competing microbes should satisfy $r_{A}=r_{B}=r$ , which simplifies the probability $\alpha_{N}^{N_{A}}$ to


(3.14) $$\begin{array}{lcr} & \alpha_{N}^{N_{A}}=\frac{rN_{A}+c_{A}}{rN+c_{A}+c_{B}}\;. & \end{array}$$

Here, the probability $\alpha_{N}^{N_{A}}$ becomes linear in $N_{A}$ . This enables us to derive the nth moment of $N_{A}(N)$ , i.e. $\left\langle N_{A}^{n}(N)\right\rangle$ , which denotes the number of A microbes when the community size equals N . In particular, considering $\langle N_{A}^{n}(N)\rangle =\sum_{N_{A}=0}^{N}N_{A}^{n}(N)P(N_{A},N)$ (see equation (3.1)) leads to


(3.15) $$\begin{array}{lcr} & \langle N_{A}^{n}(N+1)\rangle =\langle N_{A}^{n}(N)\rangle +\langle \alpha_{N}^{N_{A}}[(N_{A}+1{)}^{n}(N)-N_{A}^{n}(N)]\rangle \;. & \end{array}$$

In the electronic supplementary material, section S1, we derive the first four moments, which describe the probability distribution of the stochastic variable $N_{A}(N)$ . The first four moments allow for the calculation of variance $\sigma^{2}$ , skewness γ and kurtosis κ (see electronic supplementary material, section S1), which can then be used to compute the bimodality coefficient BC [23]. According to Sarl’s definition, the bimodality coefficient is given by $(\gamma^{2}+1)/\kappa$ , which here leads to


(3.16) $$\begin{array}{lcr} & \mathrm{BC}\underset{N\gg 1}{\approx }\frac{(c_{A}+c_{B}+3r)\left(4r\left(c_{A}^{3}+c_{B}^{3}\right)+4r^{2}\left(c_{A}^{2}-c_{A}c_{B}+c_{B}^{2}\right)+c_{A}c_{B}(c_{A}+c_{B}{)}^{2}\right)}{3(c_{A}+c_{B}+r)(c_{A}+c_{B}+2r)\left(2r\left(c_{A}^{2}-c_{A}c_{B}+c_{B}^{2}\right)+c_{A}c_{B}(c_{A}+c_{B})\right)}\;. & \end{array}$$

In the case where both species have the same dispersal rate (i.e. $c_{A}=c_{B}=c$ ), the bimodality coefficient satisfies


(3.17) $$\begin{array}{lcr} & \mathrm{BC}=\frac{N(2c+3r)(2c+rN)}{\left(2c+r)(3rN^{2}+2c(3N-2)\right)}\underset{N\gg 1}{\approx }\frac{2c+3r}{3(2c+r)}\;. & \end{array}$$

Note that the dependence on N vanishes rapidly as N increases, and the expression for BC approaches a constant determined solely by the ratio r/c . The bimodality coefficient was experimentally measured by Vega & Gore [2] to relate the heterogeneity of microbiota to the microbial density present in the pool from which the hosts fed. The BC is a summary statistic of probability distributions that ranges from 0 to 1. A bimodality coefficient equal to 1 corresponds to a Bernoulli distribution, whereas a bimodality coefficient equal to 5/9 corresponds to a uniform distribution. Thus, BC values greater than 5/9 indicate a bimodal distribution, whereas BC values lower than 5/9 indicate an unimodal distribution. Assuming that the dispersal rate is much lower than the division rate (i.e. c≪r ) leads to a bimodality coefficient equal to 1, resulting in a Bernoulli distribution where the final number of A microbes is zero with probability 1/2 and N with probability 1/2 (see equations (3.8) and (3.9) with $c_{A}=c_{B}=c$ ). Conversely, assuming the opposite (i.e. c≫r ) leads to a bimodality coefficient equal to 1/3, resulting in a binomial distribution satisfying equation (3.12). Finally, assuming that the division and dispersal rates are equal (i.e. r=c ), in addition to assuming that N≫1 , yields a bimodality coefficient equal to 5/9, resulting in a uniform distribution satisfying equation (3.19) (see §3.5).

### 3.5. Neutral case—intermediate-dispersal regime

When the dispersal and division rates are equal (i.e. $c_{A}=c_{B}=r_{A}=r_{B}$ ), the probability $\alpha_{N}^{N_{A}}$ simplifies into


(3.18) $$\begin{array}{lcr} & \alpha_{N}^{N_{A}}=\frac{N_{A}+1}{N+2}\;. & \end{array}$$

Here, the probability satisfying


(3.19) $$P(N_{A},N)=\frac{2}{N+1}\;$$

is a solution of equation (3.1). In this case, $P(N_{A},N)$ is independent of the number of A microbes, and the structure of microbial communities follows a uniform distribution.

## 4. Results

### 4.1. The relationship between dispersal and division drives the assembly of microbial communities

In our model, a new community is assembled by microbes from a microbial pool in the environment. In Vega & Gore’s experiment [2], in which worms feed on bacteria, the dispersal rate depends on the bacterial densities in the microbial pool. A high density of microbes induces a high dispersal rate and vice versa. Once inside the community, microbes divide and compete, populating the microbiota. The community assembly is thus composed of two processes: dispersal and division. For simplicity, we initially consider a neutral case in which the two microbial species have the same division rate and dispersal rate (i.e. $r_{A}=r_{B}=r$ and $c_{A}=c_{B}=c$ ). The time between two dispersal events of microbes that establish in the microbiota, denoted by $T_{c}$ , is equal to the inverse of the dispersal rate, i.e. $T_{c}=1/c$ . The growth time of a microbial community starting from an individual until the carrying capacity is reached, assumed without any microbial dispersal, is given by $T_{r}=\sum_{N=1}^{K-1}1/[r(1-N/K)N]\underset{K\gg 1}{\approx }2\;\ln(K)/r$ . This expression for the growth time assumes logistic growth and is obtained by summing the average of each division time. Both timescales are equal if and only if $T_{c}=T_{r}$ . Defining $c_{\lim}$ as the dispersal rate for which this equality is satisfied, we obtain


(4.1) $$\begin{array}{lcr} & c_{\lim}=\frac{r}{2\;\ln(K)}\;. & \end{array}$$

Thus, as in Vega & Gore’s work [2], we expect community assembly to be driven by microbial dispersal if $c>c_{\lim}$ , by microbial dispersal and division if $c∼c_{\lim}$ , and by microbial divisions if $c<c_{\lim}$ . However, since Vega & Gore [2] focused on the exponential growth phase, they found $c_{\lim}=r$ .

### 4.2. Dispersal and division timescales shape diversity within and between communities

**Figure 2.**
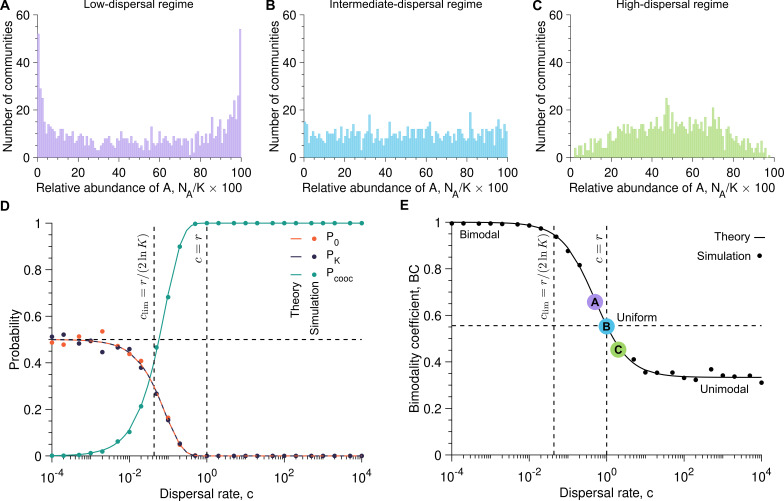
Our analytical predictions capture distinct microbial assembly regimes and diversity patterns. Panels (A), (B) and (C) show the number of communities versus the relative abundance of A microbes once the carrying capacity is reached ( $N_{A}/K\times 100$ ) with the dispersal rate equal to c=0.5 , c=1 and c=2 , respectively (here $c_{A}=c_{B}=c$ ). Panel (D) represents the probabilities that, at the end of the microbial community assembly, the community is left with no A microbes ( $P_{0}$ ), has only A microbes ( $P_{K}$ ), and is composed of both species ( $P_{\mathrm{cooc}}=1-P_{0}-P_{K}$ ) as a function of the dispersal rate c . Panel (E) presents the bimodality coefficient BC against the dispersal rate c . In panels (D) and (E), the solid lines show analytical predictions (equations (3.4)–(3.6) and (3.17)), whereas the markers correspond to simulated data averaged over $10^{3}$ stochastic replicates, each considered a community. In panels (D) and (E), the vertical dashed lines show the dispersal rate value at which the timescales of dispersal and division are equal ( $c=c_{\lim}$ ; equation (4.1)), as well as the point where the dispersal and division rates are equal ( c=r ). In panels (D) and (E), the horizontal dashed line shows a probability of 1/2 and a bimodality coefficient of 5/9, respectively. Parameter values: division rates $r_{A}=r_{B}=1$ , carrying capacity $K=10^{5}$ , number of communities $10^{3}$ .

We first focus on two microbial species that neutrally compete. In other words, both microbial species have the same dispersal and division rates (i.e. $c_{A}=c_{B}=c$ and $r_{A}=r_{B}=r$ ), which allows us to assess the impact of dispersal and division timescales on community assembly. More specifically, we consider the cases in which microbes divide more often than disperse (i.e. r>c ), divide as often as disperse (i.e. r=c ), and divide less often than disperse (i.e. r<c ). Figure 2A–C shows that these three cases lead to different outcomes for the diversity within and between communities.

If microbes divide more often than they disperse (i.e. r>c ), the abundance of A microbes in individual communities follows a bimodal distribution (i.e. with two peaks; see figure 2A). The first microbe dispersing into the community divides and populates it before a second dispersal event occurs. Since A and B microbes have the same dispersal rate, both peaks in relative abundance observed in figure 2 have the same amplitude. In the extreme case in which microbes divide much more often than disperse (i.e. r≫c ), the number of A microbes follows a Bernoulli distribution (see equations (3.8) and (3.9) and electronic supplementary material, figure S2A), such that half of the communities are populated with only A microbes whereas half of the communities are populated with only B microbes. Therefore, the *low-dispersal regime* induces low diversity within communities but high diversity between communities (i.e. low α -diversity but high β -diversity; see electronic supplementary material, figures S5 and S6).

If microbes divide as often as they disperse (i.e. r=c ), the abundance of A microbes in individual communities follows a uniform distribution (figure 2B). Both division and dispersal contribute to the community assembly, resulting in numerous possible microbial structures, which are all achieved with the same probability equation (3.19). Therefore, the *intermediate-dispersal regime* induces high diversity within and between communities (i.e. high α - and β -diversities; see electronic supplementary material, figures S5 and S6).

If microbes divide less often than they disperse (i.e. r<c ), the abundance of A microbes in individual communities follows a unimodal distribution (i.e. with a single peak; see figure 2C). The microbial communities are mostly populated with dispersing microbes before any division occurs. In the extreme case in which microbes divide much less often than disperse (i.e. r≪c ), the abundance of A microbes follows a binomial distribution (see equation (3.12) and electronic supplementary material, figure S2B). This binomial distribution shows that the microbial community structure in each community reflects the dispersal rates of A and B microbes, which are here equal, thus resulting in a single peak around 50% of microbes A, and thus 50% of microbes B, in individual communities (see figure 2C). Therefore, the *high-dispersal regime* induces low diversity between communities but high diversity within communities (i.e. high α -diversity but low β -diversity; see electronic supplementary material, figures S5 and S6).

As reported in figure 2D,E, the probability of co-occurrence of both microbial species within a community $P_{\mathrm{cooc}}$ and the bimodality coefficient BC illustrate the transition from non-co-occurrence, observed when microbes divide more often than disperse (i.e. r>c ), to co-occurrence, observed when microbes divide less often than disperse (i.e. r<c ). On the one hand, as reported in figure 2D, the probability of co-occurrence $P_{\mathrm{cooc}}$ increases from 0 to 1 as the dispersal rate c increases, passing through 1/2 when the dispersal rate satisfies $c=c_{\lim}$ (see equation (4.1)), i.e. when both dispersal and division timescales are equal. Our analytical predictions, given by equations (3.4)–(3.6) allow us to predict whether both microbial species are present at the end of the community assembly and, thus, whether high diversity within communities emerges.

On the other hand, as shown by figure 2E, the bimodality coefficient BC decreases from 1, corresponding to a bimodal Bernoulli distribution and synonymous with low diversity within communities and high diversity between communities (i.e. low α diversity but high β diversity), to 1/3, corresponding to a unimodal distribution and synonymous with high diversity within communities and low diversity between communities (i.e. high α diversity but low β diversity), as the dispersal rate c increases. Therefore, in addition to predicting whether high diversity within and between communities probably occurs or not, equation (3.17) quantifies the exact shape of the abundance fluctuation distribution describing the microbial community structure.

Our results highlight how a stochastic formalism is necessary to capture the fluctuations in the abundance of A and B microbes during assembly of a new community and, therefore, to characterize diversity within and between communities. Both the deterministic and stochastic approaches predict that, on average, neutrally competing species will be present in the community at equal abundances (i.e. $N_{A}=N_{B}=K/2$ ; see electronic supplementary material, equations S1 and S11). Although our simulated data validates this prediction, it does not reflect the observed microbial community structure. In particular, figure 2A–C shows that, for every dispersal rate, the mean percentage of A microbes at the end of the community assembly is 50%. However, figure 2A reveals that very few communities are populated with 50% of A and B microbes when the dispersal rate is low. Conversely, figure 2C shows a high number of communities with 50% of A and B microbes for higher dispersal rates. The initial stochastic period in the low-dispersal regime thus crucially influences the observed abundance fluctuation distributions independent of the final community size.

### 4.3. Differences in dispersal rates lead to asymmetry in species abundances

**Figure 3.**
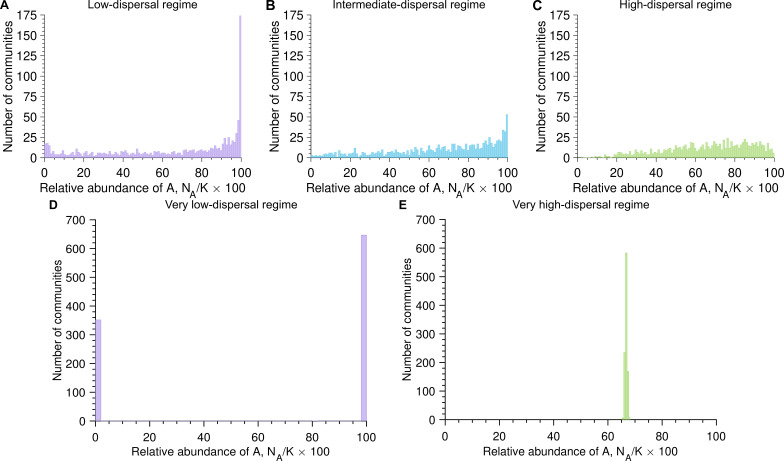
Differences in dispersal rates induce asymmetric abundance fluctuation distributions. Panels (A), (B), (C), (D) and (E) show the number of communities versus the relative abundance of A microbes once the carrying capacity is reached ( $N_{A}/K\times 100$ ) with the dispersal rate equal to c=0.5 , c=1 , c=2 , $c=10^{-4}$ and $c=10^{4}$ , respectively (here $c_{A}=2c_{B}$ and $c=(c_{A}+c_{B})/2$ ). Parameter values: division rates $r_{A}=r_{B}=1$ , carrying capacity $K=10^{5}$ , number of communities $10^{3}$ .

Next, we assume that the two microbial species have the same division rates but different dispersal rates (i.e. $r_{A}=r_{B}$ and $c_{A}\neq c_{B}$ ). As a reminder, differences in dispersal rates may result from a different relative abundance in the microbial pool or a different ability to persist in the microbial community after dispersal. Figure 3 illustrates a case in which A microbes disperse twice as often as B microbes (i.e. $c_{A}=2c_{B}$ ). Similar to the neutral case, figure 3 shows a transition from a bimodal distribution of the relative abundance of A microbes in the microbial communities, obtained for low dispersal rates compared with division rates (i.e. r>c ), to an unimodal distribution, obtained for high dispersal rates compared with division rates (i.e. r<c ). However, as opposed to the neutral case, the distribution of the relative abundance of A microbes in the microbial communities is no longer symmetric.

In the low-dispersal regime*,* figure 3A shows that a majority of communities are now populated with only A microbes. In this regime, community assembly is driven by the first microbe dispersing from the microbial pool, which is, here, twice as probable to be an A microbe than a B microbe as $c_{A}=2c_{B}$ . When dispersal rates are extremely low compared with division rates (i.e. $c_{A},c_{B}\ll r$ with $c_{A}=2c_{B}$ and $r_{A}=r_{B}=r$ ), a fraction of $c_{A}/(c_{A}+c_{B})$ communities is populated with only A microbes, whereas a fraction of $c_{B}/(c_{A}+c_{B})$ communities is populated only with B microbes (see equations (3.8) and (3.9), and figure 3D). Therefore, similar to the neutral case, the low-dispersal regime leads to no diversity within communities but high diversity between communities (i.e. low α -diversity but high β -diversity).

In the high-dispersal regime, figure 3C shows that most communities are populated with a majority of A microbes. In this regime, community assembly is mostly driven by dispersal events rather than divisions. As A microbes disperse more frequently than B microbes ( $c_{A}=2c_{B}$ in figure 3), the peak in the distribution of the relative abundance of A microbes is shifted from the centre for the neutral case (i.e. 50% ) to the right (i.e. between 50% and 100% ). In the extreme case in which microbes divide much less often than disperse (i.e. $c_{A},c_{B}\gg r$ ), most communities are populated with $c_{A}K/(c_{A}+c_{B})$ A microbes and $c_{B}K/(c_{A}+c_{B})$ B microbes (see equation (3.12) and figure 3E). Therefore, similar to the neutral case, the high-dispersal regime leads to high diversity within communities but low diversity between communities (i.e. high α -diversity but low β -diversity).

### 4.4. Differences in division rates become invisible in the very low- and high-dispersal regimes

Next, we assume that the two microbial species have the same dispersal rates but different division rates, resulting in non-zero selection coefficients (i.e. $c_{A}=c_{B}$ and $r_{A}\neq r_{B}$ , leading to s≠0 ).

**Figure 4.**
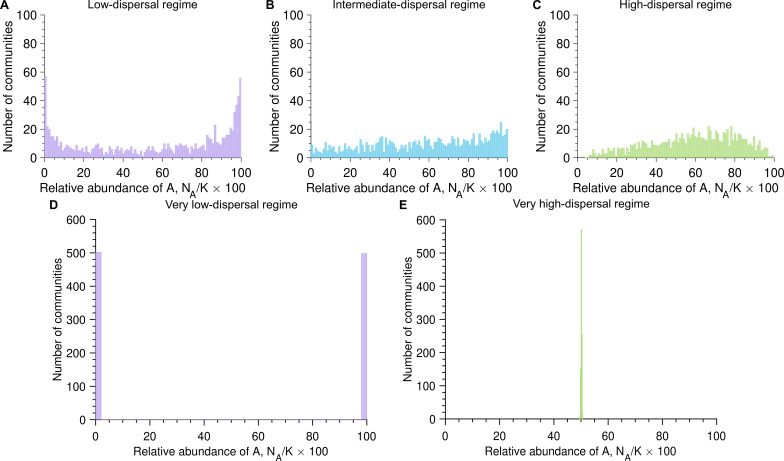
Differences in division rates do not always induce asymmetric abundance fluctuation distributions, as opposed to differences in dispersal rates. Panels (A), (B), (C), (D) and (E) show the number of communities versus the relative abundance of A microbes once the carrying capacity is reached ( $N_{A}/K\times 100$ ) with the dispersal rate equal to c=0.5 , c=1 , c=2 , $c=10^{-4}$ and $c=10^{4}$ , respectively (here $c_{A}=c_{B}=c$ ). Parameter values: division rate of A microbes $r_{A}=1.05$ , division rate of B microbes $r_{B}=1$ , selection coefficient s=0.05 , carrying capacity $K=10^{5}$ , number of communities $10^{3}$ .

Figure 4 shows simulated data for a selection coefficient of s=0.05 . In this case, A microbes divide slightly more often than B microbes, giving them a division advantage. Independent of whether microbes divide more often than they disperse (i.e. $r_{A},r_{B}>c$ ), as often (i.e. $r_{A},r_{B}\approx c$ ), or more often (i.e. $r_{A},r_{B}>c$ ), division advantage induces an asymmetry in the distribution of the relative abundance of A microbes. More specifically, we observe that most microbial communities are composed of a majority of A microbes. Therefore, asymmetry in the relative abundance of A microbes can reflect either the presence of species-specific division or dispersal advantage.

Only in the limits of very low and very high dispersal rates, division advantage plays no role in the assembly of microbial communities. As a reminder, the assembly in the very low-dispersal regime is driven by the first dispersing microbe, whereas the very high-dispersal regime is driven only by dispersal events. Thus, as shown in figure 4D,E, the distributions of the relative abundance of A microbes do not reflect the differences in division rates and are the same as in the neutral case. In other words, from communities that were assembled at very low or very high dispersal rates, it is impossible to infer whether species that seed the community are subject to within-community selection.

### 4.5. The bimodality coefficient and mean relative abundance help interpret experimental data

**Figure 5.**
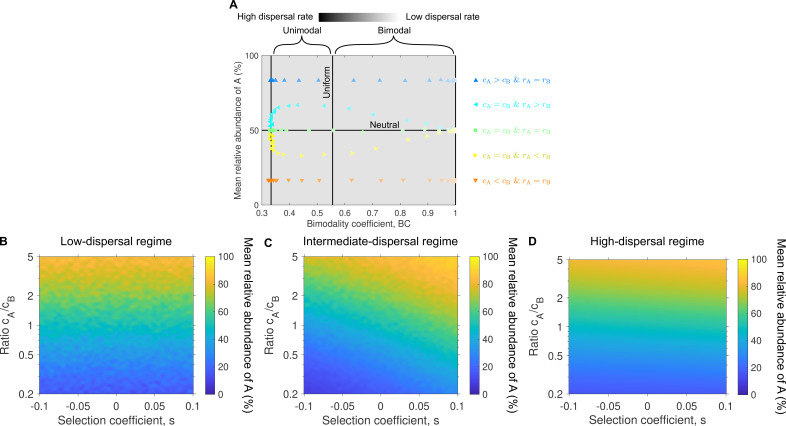
Bimodality coefficient and mean relative abundance characterize how microbial traits compare. Panel A provides a summary of our model predictions. In particular, distribution patterns characterize underlying microbial traits. We consider here the minimal case of two microbial species, namely A and B. Their dispersal rates are denoted by *c*_A_ and *c*_B_, respectively, whereas their division rates are denoted by *r*_A_ and *r*_B_, respectively. Each data point is obtained from simulating the assembly of 10^3^ microbial communities. Parameter values in panel A: division rate of A *r*_A_ = 1 (dark blue, greenish, yellow, orange) and *r*_A_ = 1.1 (cyan-blue), division rate of B *r*_B_ = 1 (dark blue, cyan-blue, greenish, yellow, orange) and *r*_B_ = 1.1 (yellow), dispersal rate of A *c*_A_ = 10^−4^ −10^4^ (cyan-blue, greenish, yellow, orange) and *c*_A_ = 5 × 10^−4^ −5 × 10^4^ (dark blue), dispersal rate of B *c*_B_ = 10^−4^ −10^4^ (dark blue, cyan-blue, greenish, yellow) and *c*_B_ = 5 × 10^−4^−5 × 10^4^ (orange), carrying capacity *K* = 10^5^, number of communities 10^3^. Panels B (*c*_A_ = 10^−4^, *r*_A_ = 1), C (*c*_A_ = 1, *r*_A_ = 1) and D (*c*_A_ = 10^4^, *r*_A_ = 1) complement the summary figure (panel A) and show heatmaps of the mean relative abundance of species A as a function of the selection coefficient s and the ratio *c*_A_/*c*_B_ in the low-, intermediate- and high-dispersal regimes, respectively. Each data point in the heatmaps represents an average over 10^3^ microbial communities. Simulation data plotted in the heatmaps are linearly interpolated. Parameter values in panels B, C and D: carrying capacity *K* = 10^5^.

Owing to the stochastic model, we identified two crucial metrics that characterize the abundance fluctuation distributions: the bimodality coefficient (or the number of peaks in abundance fluctuation distributions) and the mean relative abundance of a microbial species. Here, we argue that these metrics could be used to infer dispersal and division rates from microbial community data. Intuitively, the bimodality coefficient allows for assessing how the dispersal and division timescales compare. If the abundance fluctuation distribution has several peaks, the dispersal rates are much lower than the division rates (i.e. $r_{A},r_{B}\gg c_{A},c_{B}$ ). In contrast, if the distribution has one peak, the dispersal rates are much larger than the division rates (i.e. $r_{A},r_{B}\ll c_{A},c_{B}$ ). Second, asymmetric abundance fluctuation distributions reflect differences in dispersal or division rates. To determine which of both drives asymmetry requires data across different dispersal rates. Our analytical predictions in §3, as well as figure 5 and electronic supplementary material, figure S7, show that the signature of differences in division rates is expected to disappear at very high and very low dispersal rates (equations (3.10) and (3.13); figure 5B,D), whereas the signature of differences in dispersal rates remains visible at any dispersal rate value (figure 5B–D). When the dispersal rate of species A is higher (or lower) than that of species B, the mean relative abundance of species A exceeds (or falls below) 50%. In addition, if the division rate of species A is higher (or lower) than that of species B, it results in a maximum (or minimum) in the mean relative abundance of species A. Figure 5A and electronic supplementary material, figure S7 provide a summary of our model predictions.

It is worth noting that the mean relative abundance, unlike the bimodality coefficient, is not a diversity measure *per se* (e.g. richness or dissimilarity). However, as explained above, it plays a key role in identifying non-neutral dynamics. Deviations in mean relative abundance indicate trait asymmetries or selection, which are fundamental drivers of variation in community structure. Since selection influences diversity outcomes [14], the mean relative abundance complements traditional diversity metrics by helping to reveal the underlying mechanisms responsible for observed patterns in richness and dissimilarity.

We apply the above reasoning to data collected by Vega & Gore [2], who populated the gut of *C. elegans* worms by feeding them on a 50/50 mixture of *E. coli* bacteria fluorescently labelled with YFP or dsRed. The authors studied several mixture densities, which allowed them to quantify the microbial community structure across a range of dispersal rates.

**Figure 6.**
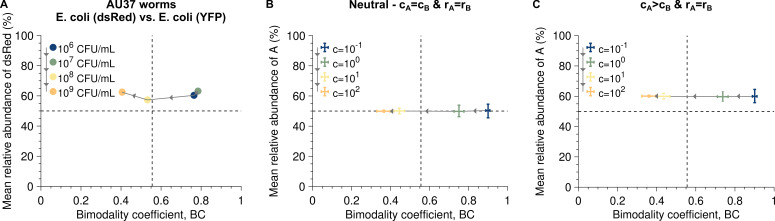
Bimodality coefficients and mean relative abundances capture a higher dispersal rate in *E. coli* (dsRed) than in *E. coli* (YFP). Panel A shows the mean relative abundance of *E. coli* (dsRed) versus the bimodality coefficient for a dataset collected by Vega & Gore [2]. Panels B and C show simulated data for the neutral case and the case where a strain has a higher dispersal rate but the same division rate as the other (i.e. *c*_A_ = 1.5*c*_B_ and *r*_A_ = *r*_B_), respectively. Each data point shows the median calculated from 10^3^ stochastic replicates of the experiment, each with 59 hosts, whereas the bars correspond to the first and third quartiles. Comparing this figure with figure 5 and electronic supplementary material, figure S7 suggests that *E. coli* (dsRed) has a higher dispersal rate than *E. coli* (YFP). Parameter values: division rates *r*_A_ = *r*_B_ = 5, carrying capacity *K* = 2 × 10^5^.

Figure 6A shows the mean relative abundance and bimodality coefficients obtained for this dataset. Consistent with the findings of Vega & Gore [2], the bimodality coefficient decreases as the bacterial density, and thus the dispersal rates, increases. This decrease reflects the transition from a bimodal to an unimodal abundance fluctuation distribution. The bimodality coefficient is equal to 5/9, which corresponds to a uniform distribution, when the bacterial density is $10^{8}$ CFU/mL. This bacterial density probably corresponds to a case in which the timescales associated with dispersal and (intra-host) division are similar.

By additionally considering the mean relative abundance, our analysis provides a complementary perspective that reveals systematic differences between strains. Although both strains are expected to neutrally compete, figure 6A suggests that the dsRed-labelled strain has a consistently higher dispersal rate than the YFP-labelled strain (see figure 5 and electronic supplementary material, figure S7). To ensure that this conclusion does not result from stochasticity due to the low number of hosts (i.e. 59), we carried out numerical simulations considering two cases. The first case corresponds to the neutral case, where each strain has the same division and dispersal rates, which could legitimately be expected here since the two strains differ only in their labels (i.e. dsRed and YFP). The second case corresponds to one in which one of the strains has a higher dispersal rate than the other but the same division rate, as figure 6A suggests. As in Vega & Gore’s [2] experiment, we considered 59 hosts and four different dispersal rates intended to represent different bacterial densities. Figure 6A,B validates that sampling stochasticity is unable to explain observed asymmetry in the data.

The observation that YFP-labelled bacteria appeared to colonize more slowly than dsRed-labelled bacteria was already noted by Vega & Gore [2] in their electronic supplementary material, figure S3. Our model puts this observation in a quantitative context; the mean relative abundance of species A when the selection coefficient is zero is given by $c_{A}/(c_{A}+c_{B})$ (see electronic supplementay material, equation S1) and, thus, depends only on the ratio $c_{A}/c_{B}$ . For the data points in figure 6A, the ratio $c_{A}/c_{B}$ is between 1.34 and 1.70. Our work implies that it is either possible that the strain labelled with dsRed has a better ability to populate a microbiota after dispersal, or that, despite the careful set-up of the experiment in [2], it may have been systematically present at greater relative abundance in the microbial pool. As previously noted by Dodge *et al.* [24], the observed difference could also arise from unequal loss of fluorescence between the two labels.

### 4.6. Our predictions remain valid at non-zero death rates and with multiple species

A first extension to our model is to include more than two microbial species, as this is highly relevant for some experimental studies (e.g. [4]) and the majority of natural microbial communities. In the electronic supplementary material, section S5, we extend some of our analytical derivations to the case of S species. For example, in the neutral case, we show that the low-dispersal regime leads to low diversity within communities and high diversity between communities such that each community is populated with only one species, proportionally to its dispersal rate (see electronic supplementary material, equation S14). Moreover, we demonstrate that the high-dispersal regime leads to high diversity within communities and low diversity between communities such that the relative abundances follow a multinomial distribution (see electronic supplementary material, equation S16). Therefore, our results on the different dispersal regimes generally remain valid when including S species. We also derive an equation giving the probability that at least two microbial species co-occur (see electronic supplementary material, equation S12, figure S3).

A second extension to our model is to include explicit death rates, which account for the natural death of microbes or for their ejection from hosts when considering host-associated microbial communities. Including a death rate is expected to change at least two features of the model. First, the final community size no longer equals the carrying capacity, but rather an equilibrium size, which, under a logistic growth, satisfies K(1−d/r) , where d is the death rate. Second, the timescale for dispersal is elongated, as the first disperser survives in the community with probability d/r . However, we expect the impact of death to be minimal if the death rate is much lower than the division rate (i.e. r≫d ). To validate this prediction, we carried out additional numerical simulations for the neutral case (i.e. $c_{A}=c_{B}=c$ and $r_{A}=r_{B}=r$ ) including a death rate d that is much lower than the division rate r (i.e. r≫d ; see the electronic supplementary material, section S6). As shown in electronic supplementary material, figure S4, our analytical predictions remain valid. The case in which the death rate is larger than the division rate (i.e. r<d ) would lead the community to decline until it goes extinct, whereas the case in which both rates are similar (i.e. r∼d ; e.g. [2]) is difficult to predict.

## 5. Discussion

Microbial communities exhibit diversity at multiple scales, both within and between communities. For example, even monozygotic human twins growing up in the same household harbour distinct microbiota [12,13]. Vega & Gore [2] demonstrated that such diversity can emerge due to stochasticity during the assembly of new communities. Specifically, they fed *C. elegans* worms a 50/50 mixture of bacteria labelled with YFP and dsRed and showed that varying bacterial densities in the mixture, as a proxy for dispersal rate, led to distinct assembly regimes, resulting in different patterns of within- and between-community diversity.

In this study, we developed and analysed a stochastic model of community assembly inspired by the work of Vega & Gore [2] and Houchmandzadeh [15]. By analysing the relative timescales of dispersal and division, our model reproduces the experimentally observed assembly regimes (see figure 2A–C): when dispersal is slower than division, assembly is dispersal-limited, resulting in high dissimilarity among communities (i.e. high β -diversity) [25]; conversely, when dispersal is faster, it homogenizes community structures, yielding lower β -diversity [25].

Our work extends previous studies in several ways. First, we derive analytical predictions for the abundance fluctuation distributions in the low-, intermediate- and high-dispersal regimes. Although these distributions may appear intuitive, our framework provides a rigorous, quantitative reasoning for their observation. Second, we calculate the probability of co-occurrence, that is, the likelihood that both species are present at the end of the assembly process, which is a key step in describing diversity patterns.

A key quantity in the description of the microbial composition across communities is the bimodality coefficient. Previously used by Vega & Gore [2] to track transitions in community structure across assembly regimes, our study provides analytical predictions of this quantity. This enables a quantitative description of how dispersal and division rates predict the emergence of within- and between-community diversity. Our results complement and extend prior theoretical work, such as Zapién-Campos *et al*. [26], who also identified distinct assembly regimes, governed by a parameter analogous to dispersal in our model. We expand on previous work by explicitly characterizing the transition between regimes.

We further investigate how trait asymmetries, i.e. differences in dispersal or division rates, influence diversity. We show that differences in dispersal produce asymmetric abundance distributions across all regimes (figure 3), whereas differences in division only manifest when dispersal and division rates are comparable (figure 4). Alongside the bimodality coefficient, we identify a second key metric: the mean relative abundance, which reflects trait differences between species. Together, these two metrics provide insight into both the assembly regime and inter-species trait differences (figure 5 and electronic supplementary material, figure S7).

To demonstrate their utility, we applied these metrics to the experimental data from Vega & Gore [2] (figure 6). Beyond supporting the previously suspected difference in dispersal rates between the two strains, our analytical approach moves past simulation-based interpretations, enabling direct estimation of the dispersal rate ratio from data.

Our analytical results and general observations remain robust when relaxing some model assumptions. They hold in the presence of non-zero microbial death rates (e.g. due to excretion or death) and extend to communities with more than two species (electronic supplementary material, sections S5 and S6). This flexibility highlights the experimental relevance of our framework and its potential use for hypothesis testing in microbial ecology.

Our model incorporates inter-specific interactions via density dependence (i.e. reduced *per capita* division rates at higher community sizes); it does not include more complex interactions such as facilitation or inhibition (e.g. commensalism, mutualism, parasitism) [27]. These interactions are known to shape microbial community structure [3,4] and warrant further exploration. Still, our model may help explain patterns observed in more complex systems. For instance, Estrela *et al*. [28] assembled replicate microbial communities from a diverse pool and observed consistent functional organization (e.g. fermenters and respirators), yet large taxonomic variation across replicates. The authors attributed this observation to early stochastic colonization and priority effects. Strikingly, our model exhibits similar behaviour under dispersal limitation (i.e. c≪r ), where early stochastic events generate high dissimilarity across communities. This suggests that stochasticity may underlie key aspects of community composition even in functionally complex and interacting communities.

In conclusion, our work advances the theoretical understanding of microbiome formation by showing how stochasticity, timescales and microbial traits collectively shape diversity both within and between communities. By offering analytical predictions and identifying key metrics, it sets the stage for testing hypotheses and interpreting experimental observations of microbial community assembly.

## Data Availability

Simulations were performed with Matlab (version R2021a). All annotated code to reproduce the simulations and visualizations is available at https://github.com/LcMrc/CommunityAssembly and has been deposited on Zenodo under the [29]. Supplementary material is available online [30].
